# A review of accident models and incident analysis techniques

**DOI:** 10.1002/acm2.14623

**Published:** 2025-02-02

**Authors:** Lawrence M. Wong, Todd Pawlicki

**Affiliations:** ^1^ Department of Radiation Medicine & Applied Sciences University of California San Diego La Jolla California USA

**Keywords:** accident model, causal analysis, safety

## Abstract

This review article aims to provide an overview of accident models and incident analysis techniques in the context of radiation oncology. Accident models conceptualize the mechanisms through which accidents occur. Chain‐of‐event models and systemic models are two main categories of accident models and differ in how accident causation is portrayed. Chain‐of‐event models focus on the linear sequence of events leading up to an accident, whereas systemic models emphasize the nonlinear relationships between the components in a complex system. The article then introduces various incident analysis techniques, including root cause analysis (RCA), London Protocol, AcciMap, and Causal Analysis Based on Systems Theory (CAST), which are based on these accident models.  The techniques based on the chain‐of‐event model can be effective in identifying causal factors, safety interventions, and improving safety.  The other techniques based on the systemic models inherently facilitate an examination of how the influence of personal conditions, environmental conditions, and information exchange between different aspects of a system contributed to an accident.  To improve incident analysis, it is essential to translate unsafe human behavior into decision‐making flaws and the underlying contextual factors. Where resources allow, it is also crucial to systematically link frontline contributions to organizational and societal aspects of the system and incorporate expertise in safety science and human factors into the analysis team.  The article also touches on related concepts such as Perrow's Normal Accident Theory (NAT), Functional Resonance Analysis Method (FRAM), and Bowtie Analysis, which are not based on specific accident models but have been used for safety improvement in radiation oncology. Overall, different incident analysis techniques have strengths and weaknesses. Taking a systems approach to incident analysis requires a shift from linear thinking to a more nuanced understanding of complex systems. However, the approach also brings unique value and can help improve safety as radiation oncology further gains complexity.

## 1 INTRODUCTION

Radiation oncology is a complex system that has evolved tremendously over the last 50 years.^1^ The growing sophistication of radiation oncology is a double‐edged sword, bringing with it both opportunities for precise treatments, and increasing complexity that poses challenges for clinicians. With the realization that accidents occur differently in complex systems compared to simpler systems, accident models and incident analysis techniques have evolved over the years. This review article has three aims. First is to describe and summarize accident models. Second is to evaluate incident analysis techniques that are used after an accident or near‐miss. Finally, this review aims to provide recommendations for improving the application of incident analysis techniques to maximize the insights generated.

One important consideration for this review is that terminology is not consistent across disciplines. Found in an international standard for occupational health and safety management systems (SMSs), an incident is defined as “an occurrence arising out of, or in the course of, work that could or does result in injury and ill health,” whereas an accident is “an incident where injury and ill health occurs.”^2^ Such delineation is similar to the distinction between an incident and an adverse event in healthcare.^3^ While the distinction between an “incident” and an “accident” is important for regulatory or management considerations of reporting and selection of safety events to analyze, those considerations are outside the scope of this review. For the purposes of this article, the terms “accident” and “incident” are used interchangeably, as the distinction between them does not affect the underlying causes or mechanisms that lead to their occurrence.

The article begins by defining the concept of an accident model, which is illustrated by a discussion of the main accident model categories: chain‐of‐event accident models, and systemic accident models. Next, techniques of incident analysis based on those accident models are introduced. Specifically, root cause analysis (RCA), London Protocol, AcciMap, and Causal Analysis Based on Systems Theory (CAST) are described. Lastly, recommendations to improve incident analysis are suggested by accounting for the strengths and limitations of the different techniques presented.

## 2 ACCIDENT MODELS

An accident model is a conceptualization of the mechanisms through which accidents occur. A variety of models have been proposed over the years as the knowledge of accident causation has evolved.^4^ Accident models are important because they are foundational to incident analysis techniques. When using an incident analysis technique, the significance of the underlying accident model can be underappreciated. In reality, the assumptions and emphases embedded in an accident model directly shape the analysis procedure, which then influences the results of the analysis.^5^

### 2.1 accident models: Domino model and Swiss cheese model

**FIGURE 1:**
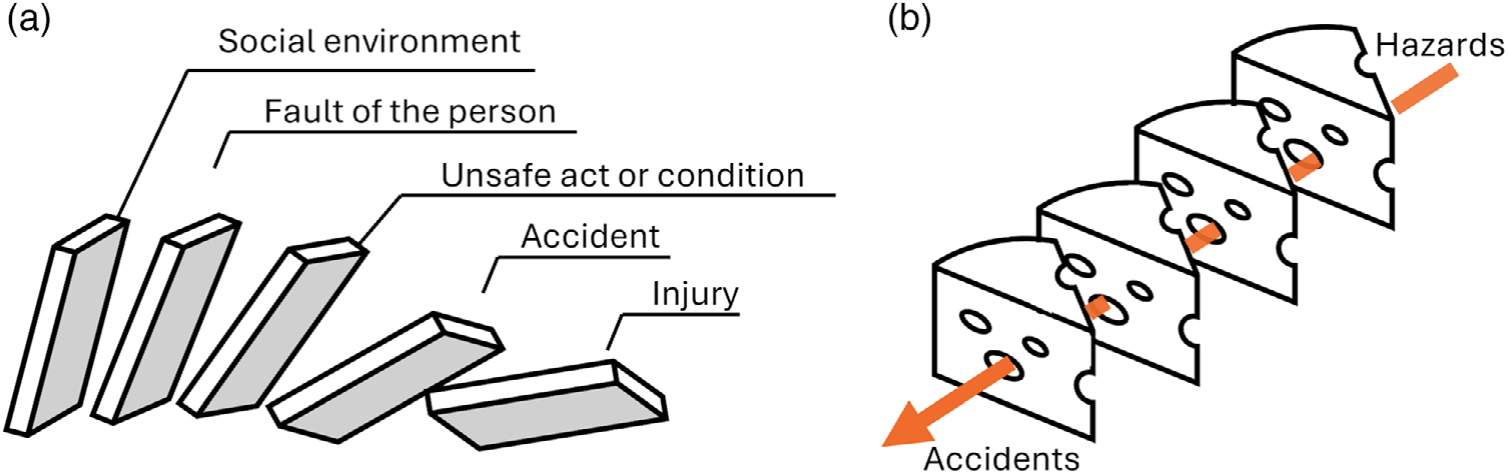
Illustrations of chain‐of‐event models. Panels (a) and (b) illustrate the Domino model and the Swiss cheese model, respectively. The salient feature is that an accident can be linearly traced to some initiating factors in the social environment or hazards. However, the dominos and the slices of cheese represent completely different causal factors.

Two well‐known chain‐of‐event models are Heinrich's Domino model and Reason's Swiss cheese model. The Domino model was proposed in the 1930s and depicts the accident process as a series of five falling dominos (Figure 1a).^6^ Except for the first domino, the other four dominos in this model primarily target the person as the cause of accidents. The first domino, “social environment and ancestry,” represents conditions outside the person's control but hampers their ability to perform (e.g., inaccessibility to education). The second domino, “fault of the person,” captures the characteristics of the person that contributed to the incident (e.g., being forgetful). The three following dominos capture the “unsafe act or condition” created by the person, the “accident” itself, and the effect of the accident, “injury.” Addressing any of the represented causes, or figuratively taking away any of the dominos, is the key to accident prevention. The heavy focus on human contributions to accidents in this model reflects its foundation in occupational health and safety and the culture of the time around the second industrial revolution—characterized by scientific discovery, mass production, and standardization.^7^

Over half a century later in 1990, another metaphor for accident causation was developed, namely, the Swiss cheese model.^8^ The model recognizes that there are many safety barriers in a system, ranging from decisions made by management to frontline tools used by workers, such as checklists. But the barriers are imperfect. The imperfections are likened to holes in the slices of cheese slices (Figure 1b). Imperfect barriers that are closer to the accident are termed “active failures.” Unsafe acts performed by frontline staff are one example. Imperfect barriers may also be deeper in the system, such as equipment design flaws, resource shortages, training gaps, and so forth. These are termed “latent conditions.” The model further recognizes that the system changes constantly, that is, holes continue to open, close, and shift. When the holes align between all slices of the Swiss cheese, an accident will occur. Accident prevention requires addressing the imperfections in safety barriers and figuratively blocking the constantly changing holes. Adding barriers (i.e., defense‐in‐depth, akin to adding slices of cheese) may be effective as well.^9^ Targeting the latent conditions is especially important because the same latent condition (flaws in the training program, for example) can manifest in different ways on the front line. That is, different active failures like mispositioning of the patient or errors in anatomy segmentation can result from the same latent condition. Therefore, targeting latent conditions can be more effective than targeting active failures.

Both models depict accident causation as a linear process, which is the hallmark of chain‐of‐event models. However, there is a major difference between the Domino model and the Swiss cheese model. The slices of cheese in Reason's model represent completely different causal factors than the dominos in Heinrich's model. Whereas the Domino model originates in occupational health in the 1930s, the Swiss cheese model captures the additional learning from major industrial accidents such as the Chernobyl disaster in 1986.^10^ The Domino model centers the causal inquiry on frontline staff without considering the broader system (beyond the social environment and ancestry). In contrast, the Swiss cheese model places a great emphasis on the contributions of the system. Specifically, the decisions unrelated to the frontline staff (latent conditions) form a critical aspect of accident causation. Therefore, effective safety management means taking strides toward addressing the latent conditions and making reforms to the organization (system) rather than targeting individual workers.^11^

### 2.2 Systemic accident models: Risk management framework (RMF) and systems‐theoretic accident model and processes (STAMP)

Rasmussen's RMF and Leveson's STAMP are two systemic accident models.^12^, ^13^ These models are called systemic models because they are developed specifically to conceptualize accident causation in complex systems. They emphasize the nonlinear, socio‐technical nature of accidents and the use of systems theory in accident conceptualization and prevention.

Rasmussen developed RMF in 1997 after having examined how risk management is undertaken in various sectors of society.^13^ His research revealed that different areas of society employ distinct risk management practices. For instance, at the individual level, human factors are important, involving topics like human‐machine interface, at the organizational level, risk management is addressed through management science, and at the societal level, law and political studies play a significant role. However, he realized that a general accident model cannot be built by simply combining the models and paradigms from individual practices. Therefore, RMF was proposed as a unified cross‐disciplinary approach.

**FIGURE 2:**
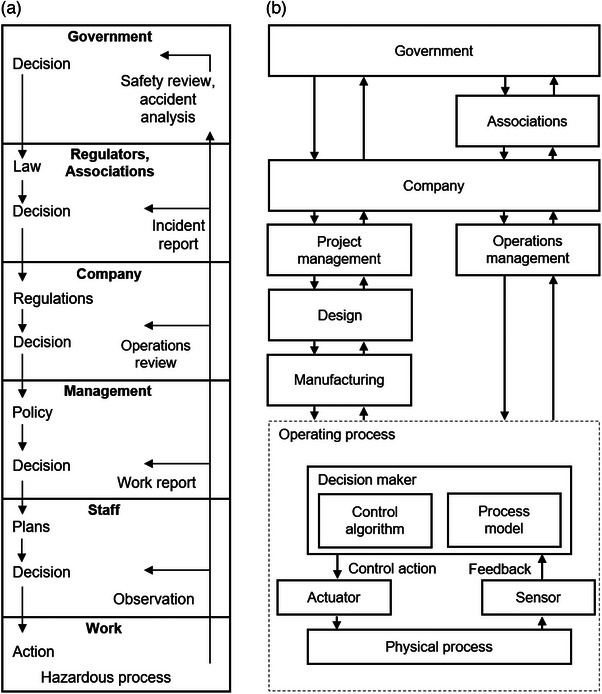
Illustrations of systemic models. Panel (a) shows a version of how accident causation and management are modeled in the RMF. Accident prevention results from the collective actions of all levels of the system. Panel (b) illustrates a similar multi‐level conceptualization in STAMP. The downward and upward arrows depict control actions and feedback, respectively. RMF, risk management framework; STAMP, systems‐theoretic accident model and processes

Rasmussen also observed a natural tendency for human behavior and organizational behavior to drift toward the boundary of safe performance.^13^ It results from an ongoing pursuit of efficiency, which prioritizes minimizing individual effort and organizational costs. While individual decision‐makers strive to optimize their behavior within their local context, the collective outcome is a gradual degradation of safeguards. Their actions ultimately cross the boundary of safe performance, which results in an accident. Based on these observations, RMF was proposed and is one of the first models where accident causation is conceptualized as a control problem, spanning multiple levels in a hierarchical fashion from government to organization to the front line (Figure 2a).

Proposed by Leveson in 2004,^12^ STAMP is another accident model that has its foundation in systems theory. Similar to RMF, the fundamental consideration in the STAMP model is that accidents result from collective actions over many levels of the system, encompassing components such as software, hardware, and workers (humans). Furthermore, Leveson highlights that safety is not a property of the individual components but is an emergent property of the system. That is safety results from the *interactions* between various components but not the *behavior* of the components alone. STAMP advances the conceptualization of accident causation by emphasizing that a focus on failures is not sufficient to fully understand accident causation. Failures such as equipment breakage or malfunction are problems that occur at the component level, but not all failures lead to accidents even though accidents can still occur when there are no failures. Preventing accidents is then equivalent to preventing both unsafe interactions of system components and critical component failures.

A fundamental principle of systems theory states that any system susceptible to external disturbances must have close‐loop control in order to achieve its intended purpose.^12^, ^14^ From a safety perspective, absence of close‐loop control would allow a system to drift from safe operation. This principle is used in STAMP to model accident causation. Close‐loop control is achieved by the following conditions of the system components: (1) appropriate goals that describe the desired system state or component attributes, (2) the ability to change the state of the system, (3) a representative model of the system, so that the appropriate control action can be performed, and (4) the ability to ascertain the state of the system. Only once these four conditions are met for the components (decision makers) in the system can control be established for the system as a whole. With close‐loop control, the system can dynamically adapt to external disturbances and changing conditions. In the opposite case where these conditions are not met, control becomes inadequate, and an accident can occur.

The concept is visually represented in a safety control structure in the STAMP model (Figure 2b). In a safe system, safety constraints are prescribed for the system as a whole and then defined in a top‐down fashion for each level of the system. To fulfill these safety constraints, the decision‐makers at each level use control actions to shape the behavior of the lower‐level subsystems. The decisions are based on relevant decision rules and an understanding of the situation (control algorithm and process model, respectively), which is informed by the instructions from a higher‐level decision maker as well as feedback, which captures the state of the lower‐level subsystems. Coordination with other decision‐makers at the same level may be required as well. Where flaws exist in the interactions (i.e., the arrows between the boxes in Figure 2b), an accident can occur.

### 2.3 Similarities and differences between chain‐of‐event and systemic accident models

Recognition of the wider societal influence on accident causation has been in place since at least Heinrich's Domino model.^6^ Reason has also metaphorized the dynamic aspect of systems as the opening and closing of the holes in Swiss cheese.^15^ These considerations embody important concepts in systems thinking and are shared between the chain‐of‐event and systemic models.

Where the chain‐of‐event and systemic models differ is the emphasis on a nonlinear causal relationship in the systemic models. The value of the nonlinear characterization of accident causation can be better understood in terms of the emphasis on context in addition to the action or event itself, and adopting a functional, rather than temporal, perspective. To elaborate, context is the influence of personal conditions, environmental conditions, and information exchange. Not only are actions and decisions heavily influenced by context, but the same action or decision can have completely different safety implications under different contexts. For example, treating a patient in the same radiotherapy clinic has different risk profiles when that clinic is overly busy versus when the clinic is operating at a routine pace. The focus on context helps prevent hindsight bias—the tendency to overestimate one's ability to have foreseen the incident^16^—to facilitate a more meaningful understanding of the incident to be developed.

Accident causation is also centered on the functions of the components in the systemic models rather than on the antecedent‐descendent relationships of the events and actions. Focusing on functions is a way to allow the interactions between different aspects of a system to be examined. In contrast, an event‐based perspective focuses primarily on the causal factors that are most visible, which tend to be proximal to the accident, and those on the front line where the result of the accident is obvious. With this uneven distribution of attention, some crucial functions that are not immediately apparent might be overlooked, despite their importance in understanding the accident.

**FIGURE 3:**
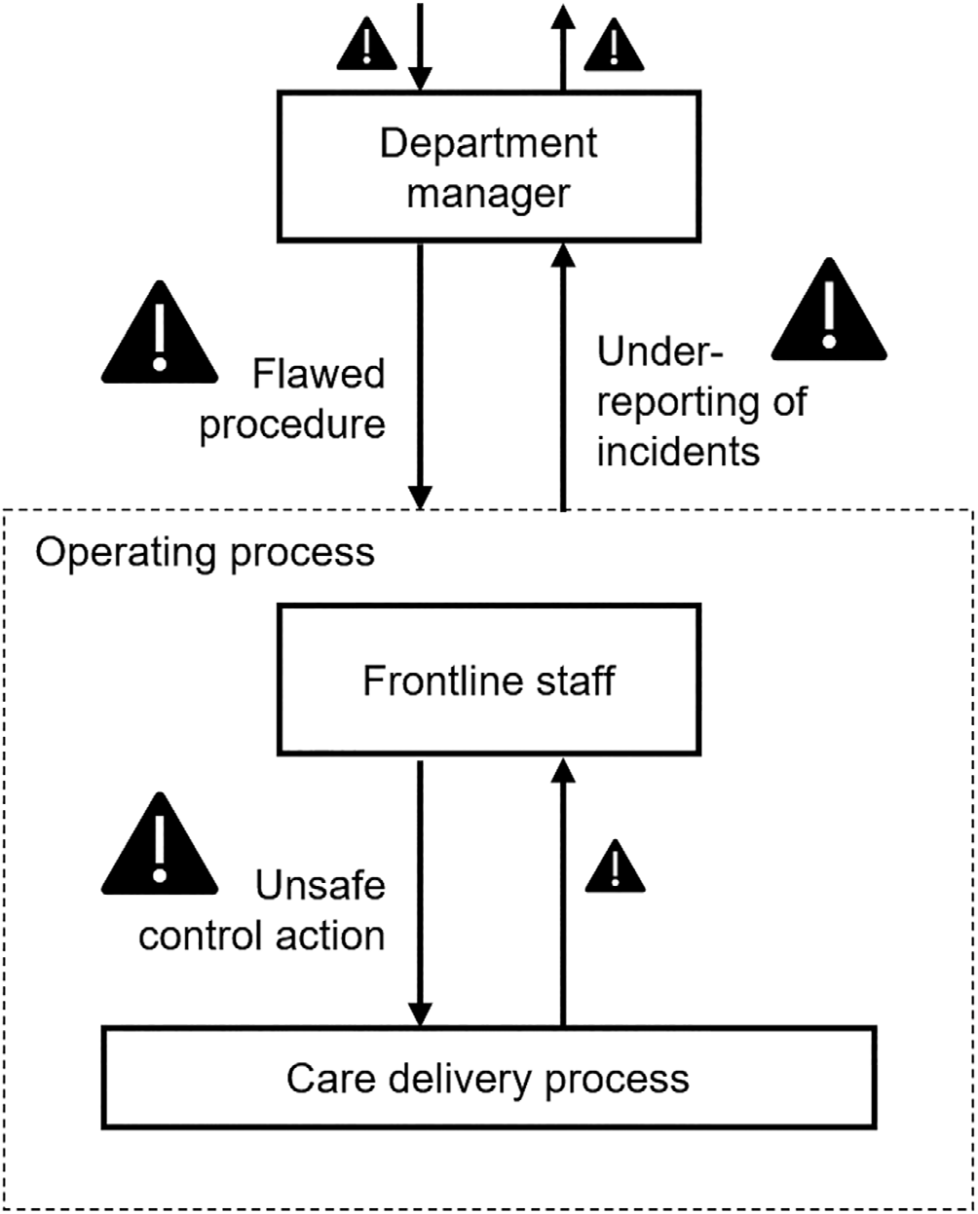
An interactive multilevel understanding of accidents is supported by the systemic models. In this example, an unsafe control action on the front line can be traced partly to the flawed procedure developed by the higher level. However, the flawed procedure arose also partly due to the under‐reporting of related incidents from the front line. The smaller warning symbols depict additional contributing factors in other parts of the system.

Combined, the functional view of accident causation in the systemic models and the focus on context culminate in a nonlinear characterization of accident causation. In each level of a system, system functions are concretely captured in control actions. Additionally, feedback captures an important aspect of the context. This construct allows any lower‐level (e.g., frontline) contributions to be transparently and systematically connected to the higher system levels (e.g., organizational and societal factors). At the same time, unsafe decisions in higher levels of the system can be understood based on the context, which encompasses the information, or lack thereof, from the lower levels. For example, an unsafe act by frontline staff can be related, in part, to a flawed operational procedure developed by departmental management. But the flawed procedure can also be traced, in part, to under‐reporting of related unsafe acts by the front line (Figure 3). The intrinsic ability to build an interactive multilevel understanding of accidents is missing from the linear models. Relatedly, one of the limitations of process maps as a part of safety analysis is that the arrows connecting each step encapsulate a lot of essential information about system operation but explicit documentation of that information is not available to the analysis team.^17^

In fact, Reason has recognized the need for, and the challenges of, taking a nonlinear view of accident causation in complex systems.^11^ The nonlinear characterization of accident causation explicit in the systemic models clearly distinguishes them from the chain‐of‐event models. With accident causation being realistically characterized under the nonlinear construct, a more comprehensive set of causal factors is likely to be identified. Moreover, the potential for blame is reduced by explaining any human contributions to incidents in terms of the influence of the context surrounding the incident.

Besides understanding accident causation, the differences between the models have additional implications for designing and selecting safety interventions. As mentioned, adding redundant safety barriers—taking a defense‐in‐depth approach—would be a logical choice based on the chain‐of‐event models because accident prevention requires addressing the imperfections in safety barriers and figuratively blocking the constantly changing holes. However, a systemic view of accident causation highlights the limited applicability of that strategy. This is because when a redundant safety barrier prevents an accident, the fact that the primary safety barrier was breached rarely gets noticed in routine operations.^13^ Given the tendency for behavior to drift to the boundary of safe performance, safety barriers degenerate over time until an accident finally occurs. The natural response based on the defense‐in‐depth philosophy is to keep adding additional barriers, which only increase complexity and encourage risk‐taking.^18^ Another reason redundancy may not be effective is that redundant barriers may fail in the same way (common‐mode failure) or they may fail because of the same cause (common‐cause failure).^19^ As an example, a second redundant plan check by the physicist can be instituted, but all physicists can be equally affected by time stress in an overly busy clinic (a common cause) and miss the same critical item (a common mode). Note that in this example, a common‐mode failure could still occur even when the causes are different (e.g., the same critical item is missed but one physicist missed it due to time stress while another physicist missed it due to a confusing display of information). The use of multiple barriers does not necessarily enhance protection, as they are susceptible to common‐mode and common‐cause failures. Based on the systemic models, redundancy is only useful to address accidents that are caused by component failures, but accidents in complex systems primarily occur due to unsafe component interactions, not only component failures.^12^ Ultimately, directly addressing the diverse and detailed causal factors identified based on the systemic models is crucial. Some design strategies to consider include substitution of materials, simplifying the system, and ensuring observability of unsafe or unanticipated conditions.^19^, ^20^

## 3 INCIDENT ANALYSIS TECHNIQUES

While accident models provide a conceptualization of accident causation, identifying the causal factors associated with each incident requires a structured process. Incident analysis techniques are the methods to derive meaning, insights, and corrective actions from the gathered facts and information about an incident.

### 3.1 Techniques based on chain‐of‐event models: RCA and London Protocol

RCA is a well‐known incident analysis technique to identify causal factors. Its original implementation in healthcare in the 1990s was not standardized.^21^ For simplistic analyses, brainstorming and “5 whys” were used, while a more sophisticated RCA process encompassed steps to identify (1) incident timeline and associated facts, (2) active failures, and (3) latent conditions.^22^, ^23^, ^24^, ^25^, ^26^ Practitioners often oversimplified accident causation by searching for a single cause or a limited set of factors, a pitfall known as “root cause seduction.^27^” Organizational support to combat blame such as insulating the analysis team from interpersonal tensions was not in place.^28^ Based on a review of RCAs over multiple years, it is not uncommon for the analyses to stop at human errors without further identifying the underlying factors, which hampers the effectiveness of the resultant safety interventions.^29^, ^30^ Therefore, its application does not always generate useful safety learning.^31^, ^32^

To address these issues, continuous refinement of RCA has been undertaken by the National Center for Patient Safety of the Department of Veterans Affairs (VA) with the latest update published in 2021.^33^ After chartering a team and conducting just‐in‐time training, the analysis process is staged as follows:

1. Create the initial flow diagram
2. Craft the initial understanding
3. Identify information gaps
4. Use triage questions
5. Collect resources and prepare for interviews
6. Conduct the safety investigation
7. Create the final flow diagram
8. Create the cause and effect diagram
9. Craft the final understanding
10. Develop action statements

A diagram to document the sequence of events is created in Stage 1, and a narrative to describe and supplement the diagram is constructed in Stage 2. In Stages 3 to 6, fact‐finding is performed. Unresolved questions are tracked and triaged, so the subsequent investigation focuses on the system, rather than people. The unresolved questions are answered through interviews, observation, and so forth. Finally, the contributing factors, root causes, and action plans are identified and described through flow diagrams, cause and effect diagrams, and associated narratives. Importantly, it is suggested that the analysis follows rules such as each human error has a preceding cause, and procedure violations are not root causes but must have a preceding cause.

The London Protocol is another technique introduced in 2004.^34^ The Swiss cheese model serves as its foundation, so the characterization of accident causation and the analysis steps are not a drastic departure from the VA RCA process. It is a technique developed and refined, particularly for application to healthcare. The Protocol stresses the importance of avoiding simplistic explanations for accidents by emphasizing that they often have multiple contributing factors rather than a single or limited number of root causes. Furthermore, the ultimate goal is to leverage the analysis to identify systemic gaps, looking beyond the incident itself to inform forward‐thinking solutions.

The analysis stages in the London Protocol are as follows:

1. Organization and data gathering
2. Determine incident chronology
3. Identify care delivery problems
4. Identify contributory factors
5. Making recommendations and developing an action plan

First, artifacts such as medical records are sequestered and interviews are conducted to build an understanding of the incident. Next, the incident is documented through narratives, a timeline, a flow chart, and so forth. Afterward, care delivery problems, a more general term for unsafe acts, and contributory factors are identified, with specific prompts given for the latter as described below. Finally, an action plan is created.

To enable a comprehensive analysis, a list of potential contributory factors to consider is provided. The list covers factors associated with patients, tasks and technology, staff, team, work environment, organization, and management, as well as the institution. For example, the patient factors include the condition of the patient, language, and personality. The staff factors include knowledge, physical health, and mental health. The institutional factors include the economic context and links with external organizations.

### 3.2 Techniques based on systemic models: AcciMap and CAST

**FIGURE 4:**
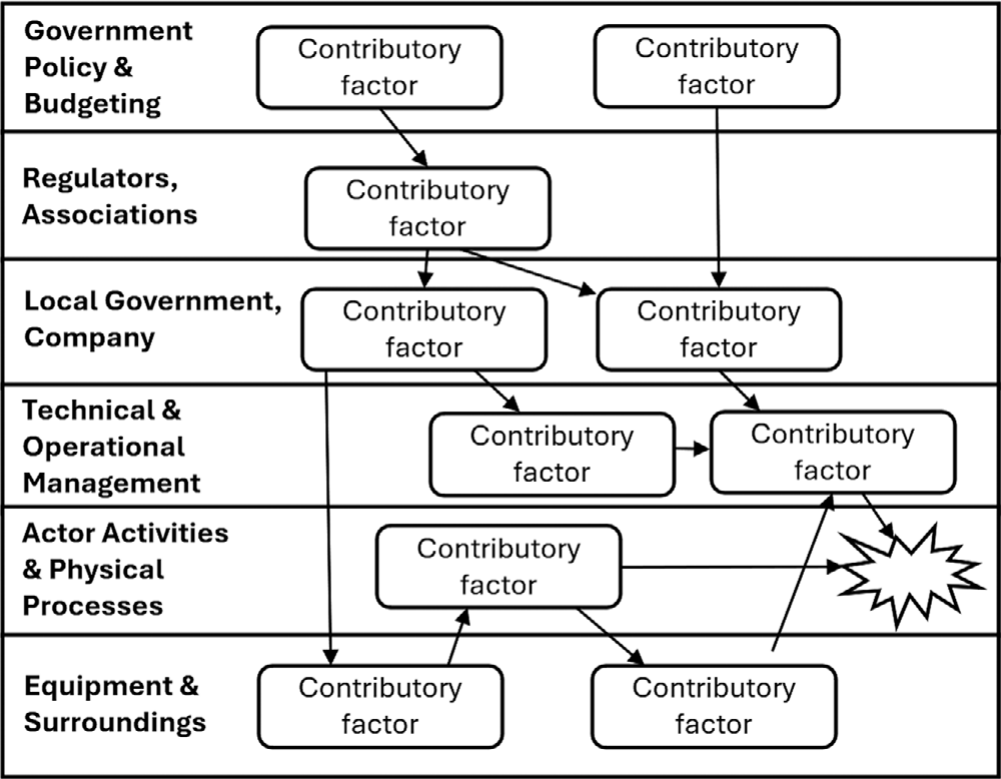
A sketch of an AcciMap.

Incident analysis techniques have also been developed based on the systemic accident models. AcciMap operationalizes RMF and provides a graphical illustration of the causal factors in an incident.^35^ The emphasis is on capturing how the decisions at different levels of a system can collectively cause an accident. An AcciMap is generically formatted into six levels (Figure 4). Causal factors are identified for each level, and the causal relationships are depicted in arrows.

When AcciMap was first introduced, only a high‐level description of the phases of analysis was described.^36^ More recently, safety researchers have proposed more detailed steps to create an AcciMap.^37^, ^38^ The latest guidelines have 10 steps:

1. Determine analysis aim and scope
2. Data collection
3. Refine hierarchical system levels
4. Construct ActorMap
5. Map the flow of events
6. Identify contributing factors
7. Place contributing factors on AcciMap
8. Identify and add relationships between contributing factors
9. Finalize and review the AcciMap diagram
10. Subject matter expert review

To elaborate on the aspects that are unique to AcciMap, Step 3 involves modifying the description in the generic AcciMap (Figure 4) based on incident details and potentially adding additional levels based on the scope of the analysis. In Step 4, an ActorMap identifies all the people and organizations that have safety responsibilities at each level of the hierarchy. In Step 7, the identified contributing factors are placed into the appropriate level of the AcciMap based on the corresponding “actor,” as identified in the ActorMap. In Step 8, the contributing factors are connected with arrows based on the cause‐and‐effect relationships. While the process does not end with a step to propose safety interventions, the need for its undertaking is implied.

AcciMap has been used to analyze adverse events in healthcare. Some past examples include medication administration errors,^39^ ingestion of superabsorbent polymer granules and the interruption of oxygen administration,^40^ medication dosing errors involving a computerized provider order entry system,^41^ and wrong patient treatment.^42^ A broad set of contributing factors is identified in each analysis, including some at the organizational level such as an organizational tolerance of missing patient identity checks and a lack of effective process to review and respond to national patient safety alerts. No analysis of radiotherapy incidents using AcciMap has been published to date.

CAST is a technique based on STAMP and has five broad steps^43^, ^44^:

1. Assemble basic information
2. Model the safety control structure
3. Analyze each component
4. Identify control structure flaws
5. Create an improvement program

Step 1 is typical to most analysis techniques, in which a list of proximal events is identified to form a basic understanding of the incident and to generate preliminary questions for further fact‐finding. In Step 2, a graphical model, safety control structure (sketched in Figure 2b), is created to capture the decision makers relevant to the incident and the interactions among them. In Step 3, each of the components is analyzed for how it contributed to the incident, starting at the physical level (e.g., equipment). Importantly, where a commission or omission of an action contributed to the incident, the emphasis is on understanding the context. Moreover, the causal factors at lower levels are traced to the contributions at higher levels to derive additional understanding. Once all the causal factors are identified for the components, Step 4 considers all of them together to check if a pattern exists. The objective is to identify systemic problems that affect multiple aspects of the system. With the problems identified both for the components individually and for the system as a whole, Step 5 generates recommendations, plans for implementation, and long‐term monitoring.

Several publications have demonstrated CAST applications in healthcare. These include analyzing a medication error involving an electronic medical record system, an incident triggering the recall of a point‐of‐care blood diagnostic analyzer, and a transplant rejection due to missed preoperative immunosuppression administration.^44^, ^45^, ^46^ Notably, an analysis of a radiotherapy incident was also performed,^47^ which is summarized as follows.

In the incident, a breast cancer patient was receiving the second fraction of external beam treatment with simple tangent fields using a surface‐guidance system. A RTT (radiation therapist) loaded the reference surface into the surface monitoring system and positioned the patient. The RTTs then performed a pretreatment timeout and started treatment. After treating the medial field, an RTT noticed the longitudinal patient position was different than what was acquired, but the displacement was less than the threshold that required action. The RTTs discussed the finding and thought that possibly the breast board was indexed incorrectly. They verified the breast board index and incline and proceeded with the lateral treatment field. After the treatment, one RTT noticed that the boost reference surface in the surface monitoring software was open and that the patient position was in fact different because the incorrect reference surface was used.

**FIGURE 5:**
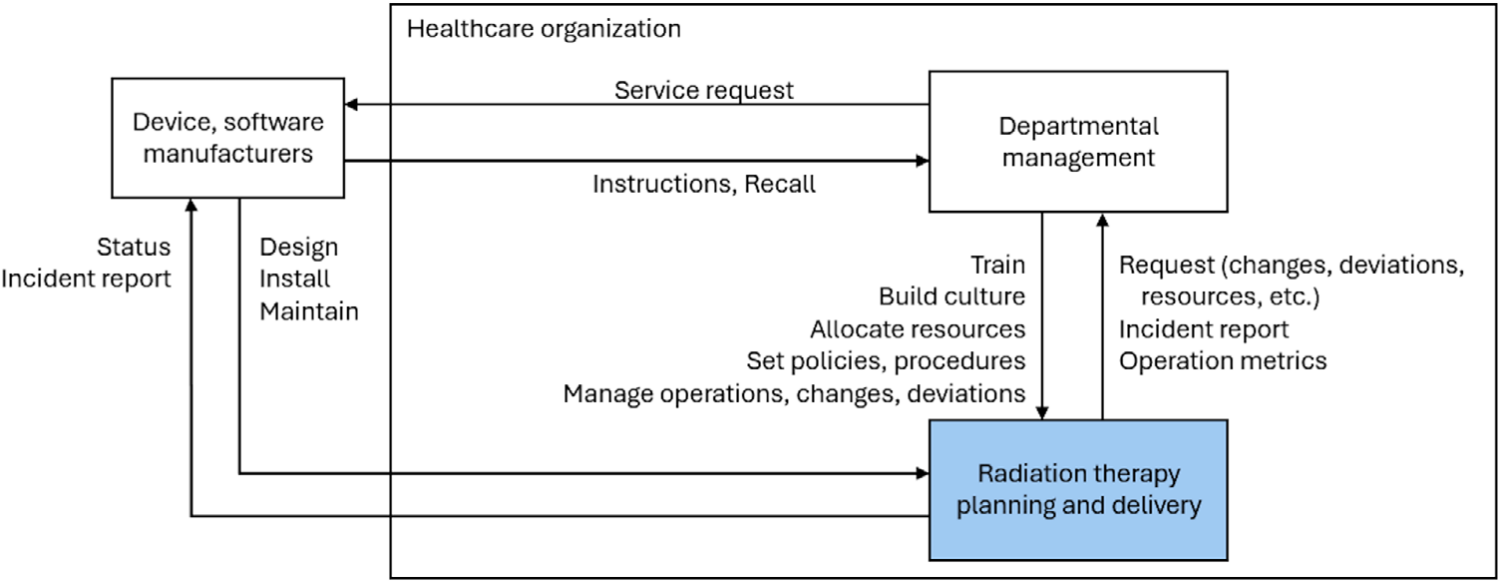
The part of the safety control structure shows the details of the healthcare organization. Color coding shows the connection between the two parts of the safety control structure.

**FIGURE 6:**
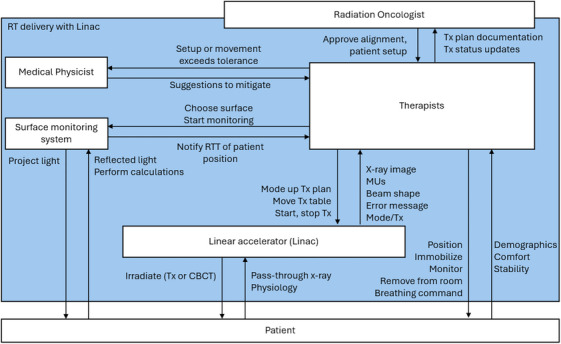
The part of the safety control structure showing the details of radiotherapy delivery. CBCT, cone beam computed tomography; MUs, monitor units; RTT, radiation therapist; Tx, treatment.

A two‐part safety control structure (Figures 5 and 6) was constructed for the analysis, and four decision‐makers were selected for examination: the RTTs, treatment planners, department managers, and manufacturers (Table 1). Beyond the causal factors associated with the individual decision‐makers, inadequate sharing of safety information was also identified as a systemic factor. It was observed that similar incidents had occurred across different centers in the enterprise, but the information was not widely discussed. Concurrently, RTTs lacked clear guidelines to troubleshoot processes adequately when potential issues were identified.

**TABLE 1:** The component analysis for four controllers.

Controllers	RTTs	Treatment planner	Department manager	Manufacturers
Contributions	Positioned the patient	Planned boost and primary treatments simultaneously without using the same isocenter	Did not set a robust procedure to troubleshoot positioning discrepancy	Produced devices with some but not full interoperability
Process model flaws	Thought the displacement was less than the threshold that required action	Thought the procedure differences were due to billing requirements	Not described	Not described
Contextual factors	The surface monitoring system indicated the patient's position to be within tolerance; should the tolerance be exceeded, the surface monitoring system would switch off the beam of the linac The boost treatment surface loaded by default Verified that the breast board index and incline were correct and acceptable	Worked at the main campus and followed procedure; provided coverage to the satellite facility remotely Frontline staff not familiar with a software update related to inactivating surfaces in the surface monitoring system	Limited radiographic imaging to minimize radiation dose to the patient Routine use of field light is not advisable due to the advent of surface monitoring systems	Not described

Abbreviation: RTT, radiation therapist.

While comparison studies between AcciMap and RCA have not been done in healthcare, comparisons between CAST and RCA do show that more causal factors are identified using CAST.^47^, ^48^, ^49^

Despite the merits, practitioners applying AcciMap and CAST have faced challenges. While AcciMap has been found to be relatively intuitive to learn by healthcare practitioners,^42^, ^50^ it can be regarded as time‐consuming.^42^ For CAST, some of the systems concepts can be unfamiliar to healthcare practitioners, and the safety control structure can be challenging to understand and create.^48^, ^50^, ^51^

## 4 EXAMPLE APPLICATION OF INCIDENT ANALYSIS TECHNIQUES

Each of the mentioned incident analysis techniques is illustrated with an abbreviated example analysis. Plausible details were synthesized to support the illustration. The emphasis is on showcasing the unique aspect of each technique rather than providing a complete analysis of the incident using a given technique.

The example analyses cover a radiation therapy incident published as an RCA case study in which a patient with rectal cancer was treated with the isocenter positioned 10 cm superior to the intended location.^52^ During simulation, the patient had refused the standard beam positioning technique with a tattoo, and instead, a nonstandard aid was used (Tegaderm over a temporary marker). The isocenter positioning landmarks were recorded in relation to the tip of the coccyx (TOC) during simulation, but the treatment chart incorrectly referenced the standard marker as the landmark. Notably, the temporary marker and TOC were separated by a distance of 10 cm. Unexpectedly, it was necessary to expedite the treatment start date. Treatment planning was completed with isocenter positioning based on the temporary marker by a planner who was not involved in the simulation. A pretreatment check of the treatment chart and plan was performed urgently and did not reveal the inaccurate instruction for isocenter positioning. On the first treatment day, the patient received radiation treatment at the same time as the arrival of an urgent ambulance patient, creating a degree of stress. During patient setup for treatment, the isocenter positioning was done based on the temporary marker, after clarification that the tattoo was not placed. The presence of the patient's family at the console also contributed to a feeling of inhibition among the staff. Mistreatment ensued because beam positioning was not done based on the TOC. The discrepancy was discovered when verification x‐rays were reviewed by two RTTs after the treatment.

### 4.1 The VA RCA process

**FIGURE 7:**
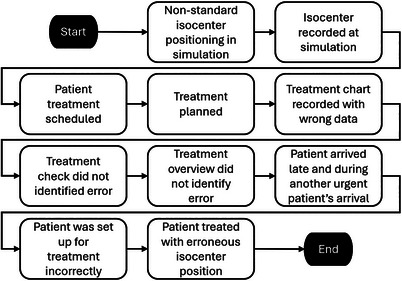
The initial flow diagram captures the incident in 10 steps, spanning from simulation to first treatment.

Figure 7 shows the initial flow diagram. The first two steps cover key aspects of patient simulation. Steps three to six span treatment planning, documentation, and checking. Steps seven to ten highlight the events just before the treatment encounter through the mistreatment.

The initial understanding resembles much of the information described when the incident is introduced at the start of Section 4. Additionally, the treatment start date was moved earlier due to a change in the patient's condition thus compressing the planning workflow. For the same reason, a different treatment planner (also an RTT in this case) not involved in the simulation took over treatment planning, which was also when the treatment chart was recorded with the wrong data. On the treatment day, the patient arrived later than the scheduled treatment time, which also coincided with another urgent patient being transported to the facility by ambulance.

Part of the information gap surrounds questions such as why was the beam positioning recorded based on the TOC even when the Tegaderm was applied, why did the pretreatment check not catch the error, and so forth.

Before conducting interviews and the safety investigation, the triage questions are considered, which enable a more expanded view of the incident. A critical dimension to examine is the options for isocenter positioning and the placement of the isocenter positioning aid or landmark. Because the isocenter positioning aid is a safety barrier to prevent inaccurate isocenter positioning, the triage questions on barriers apply. For instance, ‘Was the concept of “fault tolerance” applied in system design?’ Similarly, the triage questions on communication (e.g., “Was communication between management or supervisors and front line staff adequate?”) are relevant because the frontline staff were confronted with the difficult, nonstandard decisions surrounding the prioritization and accommodation of more than one urgent patient and the uncertainty of which isocenter positioning aid was used.

**FIGURE 8:**
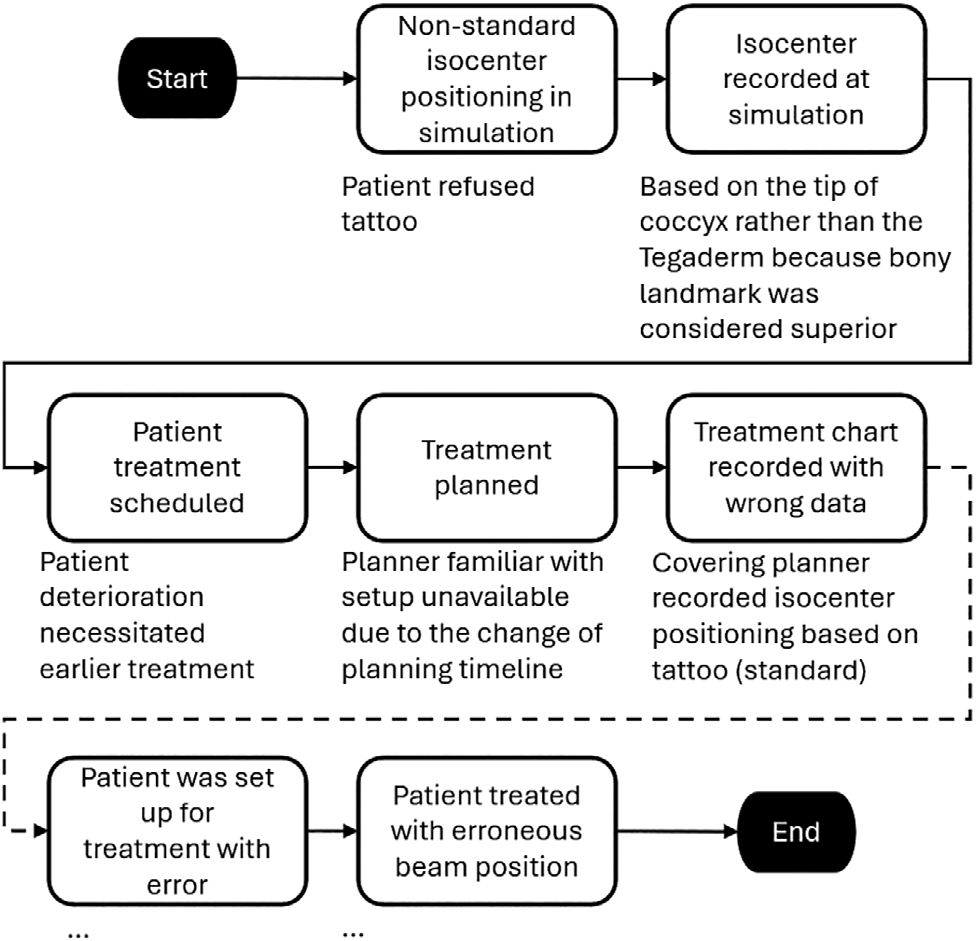
The final flow diagram captures the additional understanding gleaned in the analysis process. The diagram is abbreviated where additional steps and descriptions are not shown (dash lines and ellipses).

The information gap is then filled by interviews and investigation. Even though the nonstandard isocenter positioning aid was applied, isocenter positioning was recorded based on the TOC because the bony landmark was considered superior to Tegaderm. Because of the change in the patient's condition necessitating urgent treatment, there was substantial time pressure during treatment planning. The treatment planner involved with the simulation was also unavailable during this changed timeline, necessitating the planning to be done by a planner less familiar with the patient setup. Just before the treatment day, confusion arose as to whether the patient was going to be treated as scheduled. Therefore, the treatment overview was not completed in its entirety, and the error was not discovered. Figure 8 illustrates the final flow diagram where contextual details related to each of the steps are depicted under the step to describe the final understanding of the incident.

**FIGURE 9:**
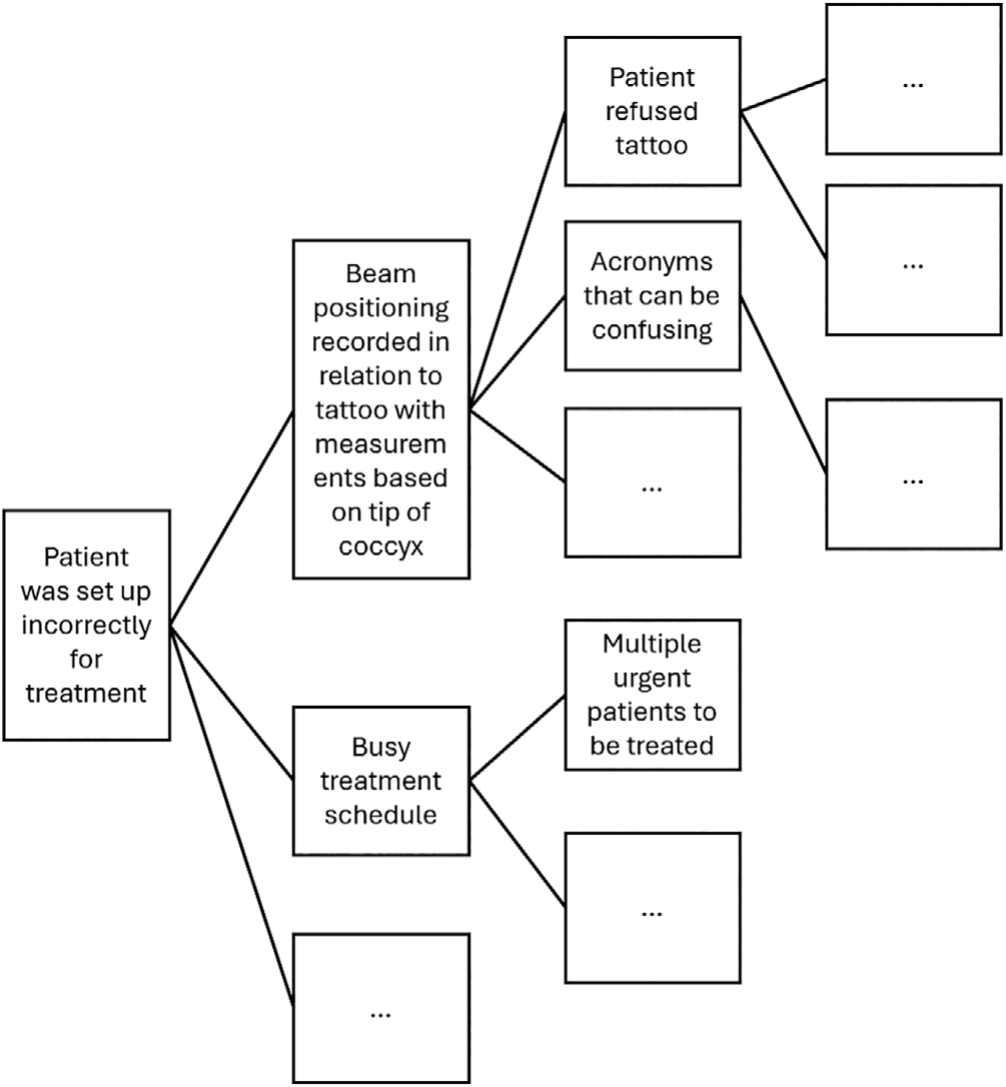
The cause and effect diagram depicts the erroneous patient setup as what should have been prevented. Each of the lines represents a “cause by” relationship. The ellipses represent additional potential reasons for why the treatment deviation could have occurred.

In the cause and effect diagram (Figure 9), the patient setup error was defined as the incident that should be prevented from recurring. The erroneous recording of isocenter positioning was a key action leading to the mispositioning of the patient for treatment. In parallel, having a busy treatment schedule also provided a condition such that the error in the documentation was not caught during the treatment overview process just before treatment. Both the documentation error and the busy treatment schedule have further underlying causes.

Importantly, answering the triage questions helps identify contributing factors beyond the front line. For instance, the administrative process to define the acceptable isocenter positioning options and their associated documentation requirement was flawed, as well as inadequate communication between management or supervisors and frontline staff when treatment involves uncertainty of which beam positioning aid was used.

### 4.2 The London Protocol

Soon after the investigation team is established, fact‐gathering begins in the London Protocol. The treatment plan, the chart, other medical records, and artifacts are sequestered. Statements are taken and interviews are performed with personnel such as the RTTs and other staff.

A flowchart such as Figure 7 and the associated narrative is used to document the chronology of the incident. Alternatively, the sequence of events could also be formatted as a list of timestamped events, such as

**30 June 9:00 AM**

The patient received a nonstandard isocenter positioning (Tegaderm)

**30 June 9:05 AM**

Isocenter positioning was recorded with reference to the TOC

The care delivery problems include isocenter positioning being erroneously recorded on the datasheet, failure to identify the problem in either the treatment plan check or the treatment overview, and failure to correctly resolve the confusion surrounding isocenter positioning during patient setup.

**FIGURE 10:**
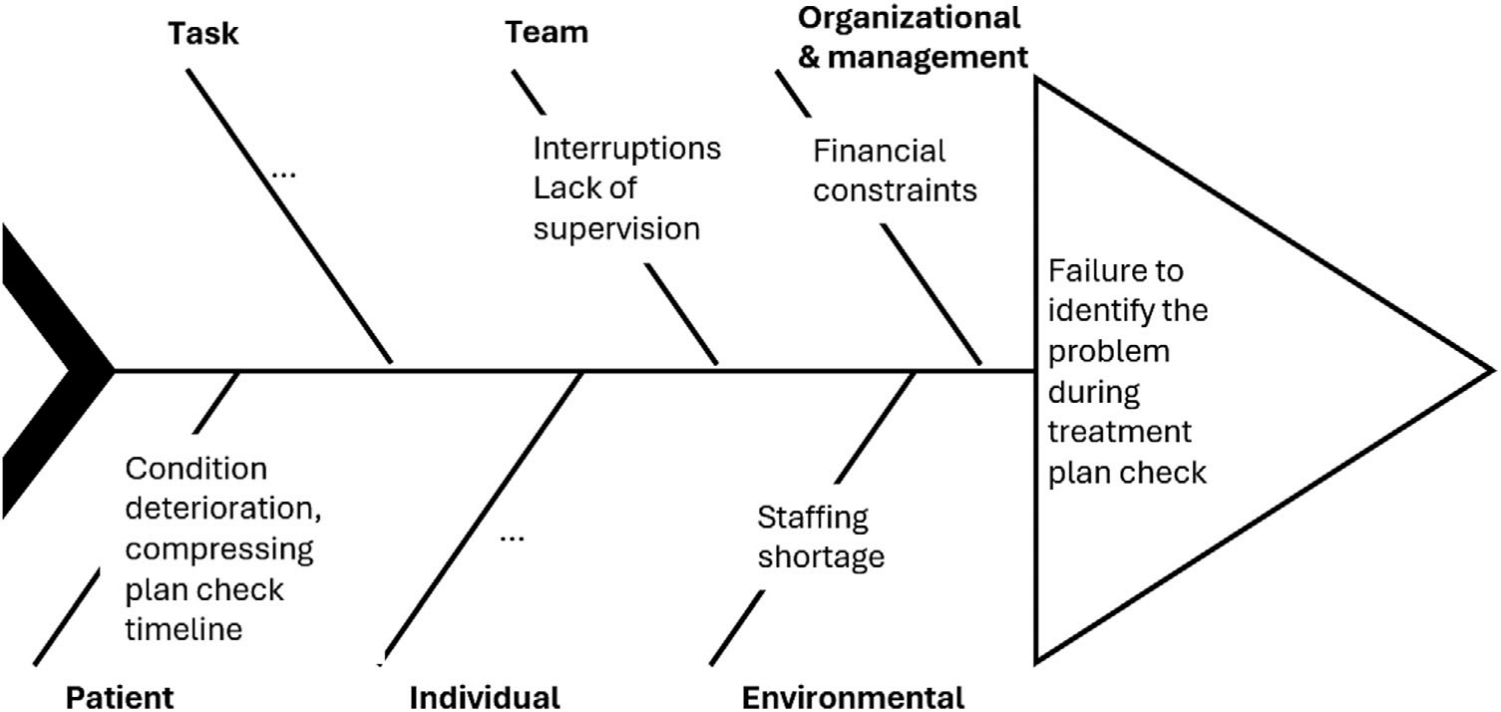
The fishbone diagram provides one option to document the contributory factors to a given care delivery problem. The contributory factors of the same type (e.g., patient factors) are consolidated in a branch of the diagram.

Each of the care delivery problems is analyzed for the corresponding contributory factors. Figure 10 illustrates the documentation of the factors associated with the failure to identify the documentation problem during the treatment plan check. Factors away from the front line, such as financial constraints at the organizational level are explicitly elicited and documented.

### 4.3 AcciMap

The aim of the analysis is to illustrate the characteristic aspects of AcciMap. Therefore, the scope of the analysis only covers a small number of decisions and actions leading up to the incident. Nonetheless, the scope covers both the front line and the management aspect of the organization.

The hierarchical system levels are modified from the generic version (Figure 4) to a combination of only three levels (top to bottom): Departmental Management, Clinical Processes, Equipment, and Surroundings. Had the aim been to more thoroughly analyze the incident, the top three hierarchical system levels could have been modified to be Government Policy and Payors; Regulators, Professional Bodies; and Hospital Management.

**FIGURE 11:**
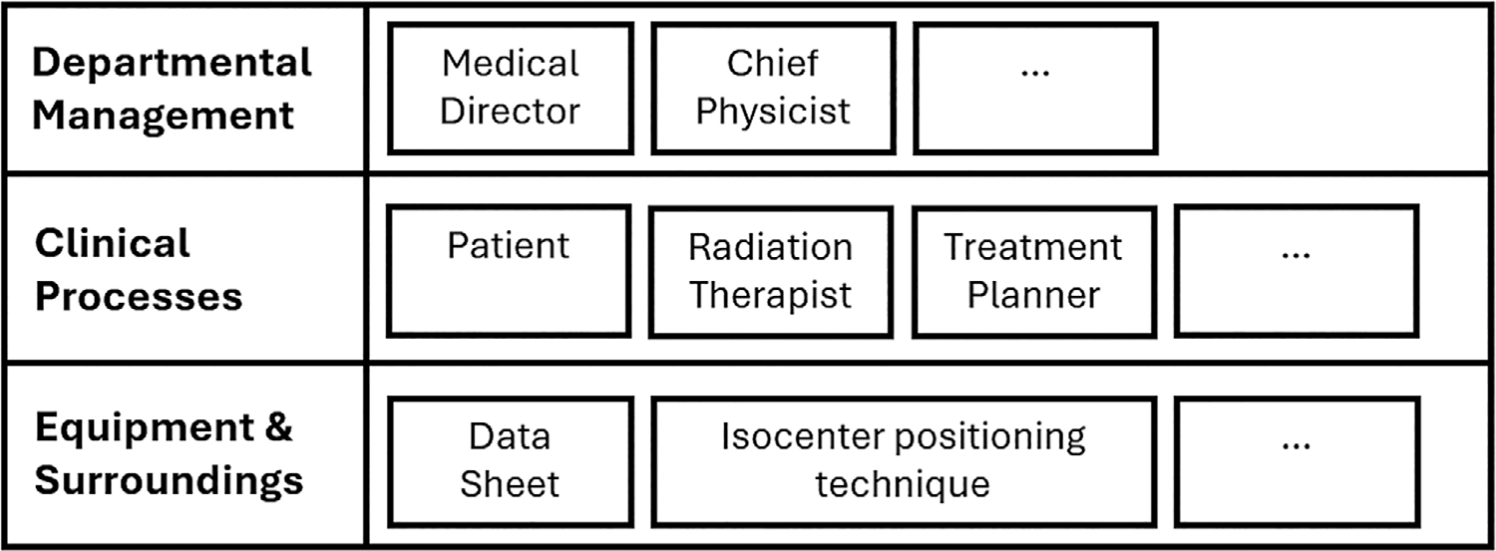
The ActorMap with an abbreviated number of decision makers in each level.

Figure 11 shows the ActorMap given the modified hierarchical system levels. In terms of communication or linkage, the medical director and chief physicist are linked to all items except the patient, with whom they do not have a direct care relationship. The patient is linked to the RTTs and isocenter positioning aid. The RTTs are linked to the treatment planner (also an RTT in this example), the datasheet, and the isocenter positioning aid.

**FIGURE 12:**
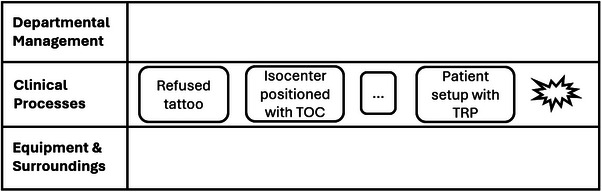
Three events are shown in this abbreviated flow of events. The explosion icon represents the final treatment deviation. The ellipses represent additional events that are omitted from this illustration. TOC, the tip of the coccyx.

An abbreviated flow of events is shown in Figure 12. Some of the events proximal to the incident include the patient refusing the tattoo during simulation, and isocenter positioning being done based on TOC. Eventually, the patient was set up with Tegaderm for the first treatment, and the treatment deviation ensued.

**FIGURE 13:**
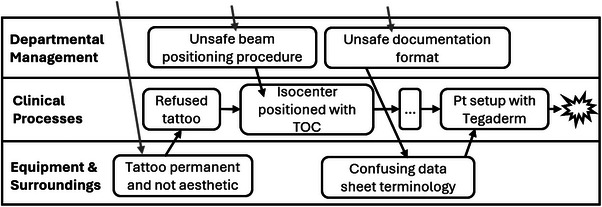
AcciMap with selected contributing factors to the incident. Dashed arrows represent causation from additional contributing factors omitted from the illustrations—those from higher levels of the system.

The contributing factors are superimposed on the flow of events (Figure 13). The refusal of the tattoo was a result of the tattoo being permanent and not aesthetically pleasing. While isocenter positioning based on the TOC was an acceptable procedure, the procedure was not paired with sufficient resources and support to be safely executed. Similarly, insufficient human factors considerations were made when establishing the data sheet to document the basis of isocenter positioning, leading to the terminology being confusing and contributing to the inconsistent patient setup and the resultant treatment deviation.

### 4.4 CAST

In this incident, the patient could have been injured from radiation overexposure at the inadvertently treated location. Correspondingly, the wrong location delivery of radiation is a hazard to be analyzed and prevented for future patient treatments. To learn from the incident using CAST, a list of proximal events like that created for the VA RCA process or the London Protocol above is a useful starting point. The preliminary questions for further fact‐finding resemble those listed for the information gap in the VA RCA process.

**FIGURE 14:**
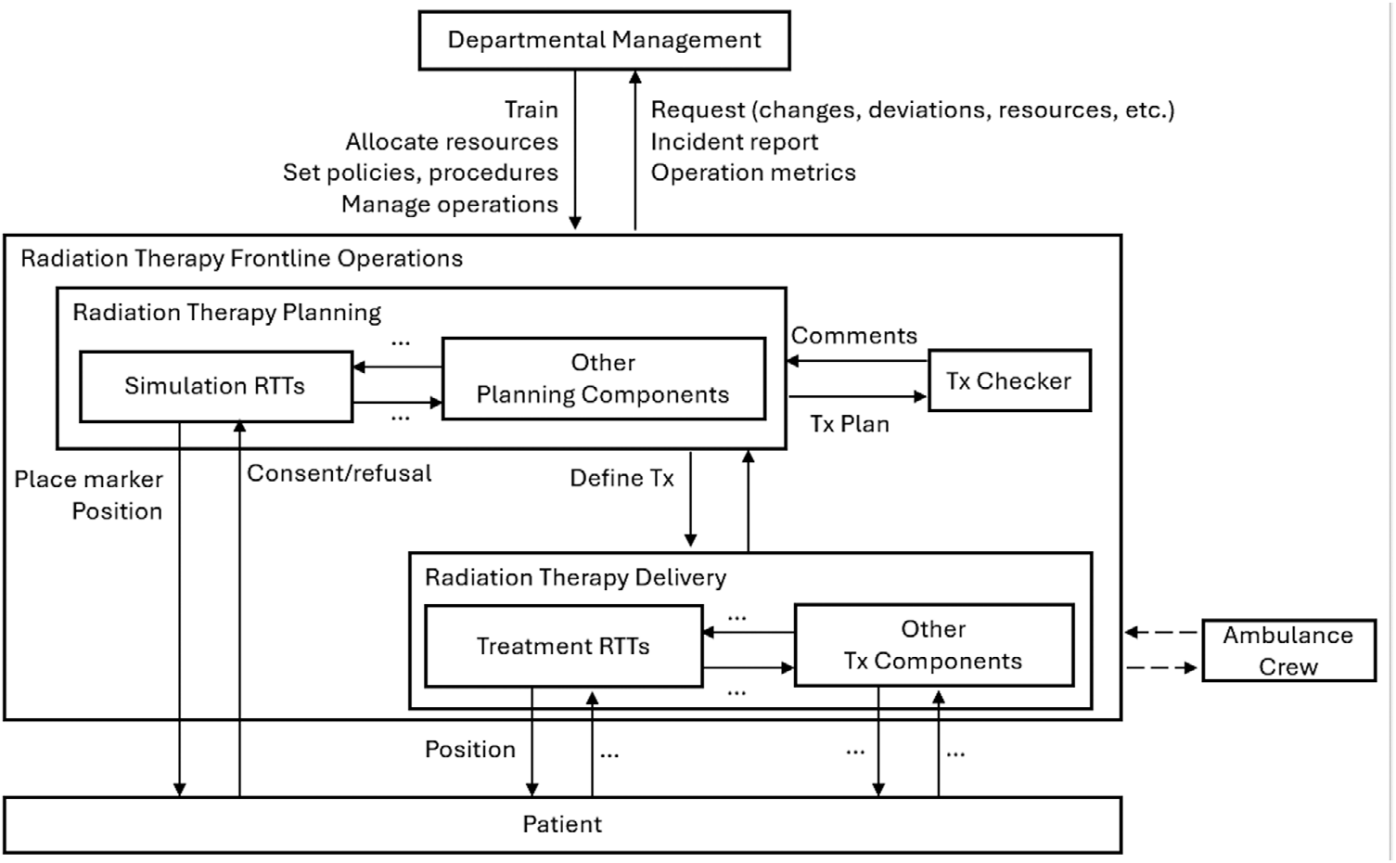
A sketch of the safety control structure for the incident. Missing interactions between the ambulance crew and treatment delivery staff that contributed to the incident are visualized by the dashed arrows connecting the boxes. Additional details are omitted from the diagram (interactions worth analyzing are encapsulated in the Other Planning Component and Other Tx Component boxes; ellipses represent additional control actions, feedback, or coordination). Tx, treatment.

Figure 14 depicts a sketch of the safety control structure. The diagram encompasses the management of the radiation therapy department, frontline operations, the patient, as well as relevant components external to the provider organization, such as the ambulance crew. Other omitted frontline components worth analyzing include treatment planners (RTTs in this case), treatment planning systems, medical physicists, radiation oncologists, and so forth.

Moving upward from the bottom of the safety control structure, no equipment failure was identified. The decisions of the treatment RTTs are next analyzed. Their primary contribution to the incident was the positioning of the patient in such a way that the treatment beam deviated from the intended location. Their process model flaw was the belief that beam positioning was based on the Tegaderm. The contextual factors underlying the process model flaw included the datasheet noting tattoos as the basis for isocenter positioning and that the treatment plan passed checking, assuaging any causes to doubt the datasheet. Some analysis questions arise here for why the datasheet was used as a record of isocenter positioning, and if a more accurate alternative was considered. These questions would be explored at the departmental management level. Before moving up to the level of treatment planning in the safety control structure, other sets of interactions in the system are worth analyzing. For example, the interactions between the ambulance crew and the treatment delivery staff. The treatment RTTs thought that the patient involved in the incident was the only urgent patient requiring treatment. This flawed process model was caused by the ambulance crew not coordinating with the treatment delivery staff prior to bringing the other urgent patient to the clinic. Notably, language such as “failure to” is not used in CAST to avoid blame.

Finally, economics is the only systemic flaw that is readily identifiable without thoroughly analyzing the contributions and the contextual factors associated with the decision‐makers in the system. Lack of management support for sufficient staffing led to a shortage at both the treatment planning and treatment delivery aspects of the system and the inability to expand capacity to facilitate simultaneous treatment of urgent patients, creating time pressure and discontinuity of the staffing in patient care. It also may have precluded the implementation of newer technologies to document and compare patient setup and isocenter positioning between simulation and treatment.

## 5 IMPROVING INCIDENT ANALYSIS

Before providing specific recommendations for incident analysis, it is important to recognize that incident analysis is only one function of an overarching SMS. In past reviews of incident analysis output, some researchers believe that SMS‐level challenges are partly the reasons why more informative causal factors and stronger safety interventions are absent.^28^, ^31^, ^53^ These challenges include a lack of resources and expertise, a lack of motivation beyond satisfying regulatory requirements, and censorship from organizational politics. Therefore, establishing organizational commitments for a system‐focused learning culture, adequate analysis resource support, and investments for corrective action implementations are important to improving incident analysis and broader safety improvement. Incorporating expertise in safety science and human factors in the analysis team is useful as well.^48^, ^54^

### 5.1 Recommendations for incident analysis based on chain‐of‐event models

While past reviews of RCA have mixed results, effective safety improvement with RCA is possible, and a careful selection and implementation of the exact RCA process is key.^53^ To that end, the use of the VA RCA process or the London Protocol can be beneficial because they are either specifically structured to overcome the shortcomings of the original RCA process or have reported evidence of safety improvement.^34^, ^55^ The gains from applying these refined techniques are also apparent from the example analyses provided above. Specifically, the published RCA identified only frontline contributions to the incident, but other contributions deeper into the system could be identified in the example analyses when the VA RCA process or the London Protocol was applied.

A direct comparison of analysis techniques is beyond the scope of this review and would require multiple analysis teams with adequate training in the respective technique, ample time for the analysis, and equal access to facts and fact‐finding opportunities. An experiment with a radiation therapy focus of this nature would be insightful especially if the outcome metrics cover not only the findings of the analysis but also support a detailed survey of the effort‐learning tradeoff.

Some additional considerations beyond the latest RCA guidance may further aid in maximizing safety learning from incidents. Unsafe human behavior should be translated into the associated decision‐making flaws and the underlying contextual factors that may have contributed to those flaws. For human decision‐makers, part of this translation is done by answering the following questions:

1. How did the decision maker choose which action to perform?
2. What does the decision maker know or believe about the situation?
3. How did the decision maker come to have that knowledge or belief?
4. What information did the decision maker have or need?
5. What other information could they have had that would have changed their decision/behavior?

These considerations improve the rigor when identifying conditions as contributing factors and directly drive the focus from human errors to the underlying factors and the dependencies on the rest of the system. For analyses of a larger scope, the need becomes even more important to *systematically* link the contributions on the frontline to the organizational and societal aspects of the system such as device design and manufacturing, operations management, regulatory bodies, best practice recommendations from professional associations, and so forth.

### 5.2 Recommendations for incident analysis based on systemic accident models

The clinical reality is that the time and resources dedicated to incident analysis are costly and the majority of the relatively frequent incidents in radiation oncology have little or no clinical impact on a patient. Therefore, scoping the analysis given the available resources is crucial. To that end, capitalizing on the strengths of the systemic models—to examine how key components interact—can be useful. Notably, Weber‐Jahnke and Mason‐Blakley have derived important insights even when their CAST analysis only covered a two‐level, frontline‐focused control structure.^45^ While the strengths of these techniques may only be fully capitalized with an elaborate analysis covering higher levels of the system, even a limited‐scale application can generate important insights not typically found using techniques based on chain‐of‐event models. For example, how a process changes and requires deviations from standard procedures (e.g., a repeat of certain control actions) and how different information input may conflict and lead to unsafe control actions.

A structured approach to facilitate AcciMap application in healthcare remains to be developed. For CAST, several initiatives are underway to improve the ease of learning and application. A prototypical safety control structure modeling radiation oncology has been developed, which can be readily adapted to any local environment.^47^ To further build proficiency in control structure creation, an introduction discussing its similarities and differences with process mapping is also available.^17^ Ultimately, building a control structure is not strictly necessary and can be omitted if there is a good understanding of the system and an effective alternative to document the system's behavior.^43^ Other initiatives include a handbook that is specifically written for the healthcare community.^43^ Training materials and templates for each step of the analysis have also been developed.^47^

### 5.3 Recommendations for identifying effective safety interventions

Once the causal factors have been identified, careful considerations are required to design and select effective safety interventions. In general, safety interventions differ by effectiveness and are categorized into taxonomies such as the safety design precedence.^4^, ^56^ To effectively eliminate the identified contributing factors, stronger interventions often require a comprehensive overhaul at the institutional level. In contrast, personnel‐level interventions, such as training, only provide individual protection and are therefore relatively weak. Particularly in the consideration of training as an intervention, the reason why prior training was not effective must be addressed before the same training should be used as an intervention. For example, simply repeating the training in response to an incident for someone that is already appropriately trained, is not addressing the problem.

On a practical level, specific tools have been proposed to improve safety intervention design and selection. In the work by Hettinger et al., categories of interventions such as information technology structure, physical environment, training, compliance checks, and so forth. have been assessed for their effectiveness and sustainability.^57^ These categories and their specific examples serve to guide the ideation process. A process is also proposed by Card et al. to design diverse intervention options, refine the options, and select the optimal options for implementation.^58^ Some key aspects of the process include eliciting intervention options specifically to improve situational awareness, providing administrative control, and so forth, and further analyzing the options for strengths and weaknesses, side effects, and costs.

## 6 RELATED CONCEPTS AND FURTHER STUDIES

Additional perspectives on accident causation exist, such as Perrow's Normal Accident Theory (NAT).^59^ In NAT, a system is classified in terms of complexity and coupling. When applying NAT, a system is considered complex when the interactions within the system have unplanned and unexpected sequences. In contrast, a system with visible and familiar interactions is considered a linear system. In terms of coupling, rapidly sequenced interactions with little room for intervention qualifies a system as tightly coupled, whereas slow interactions with ample opportunities for corrective actions are the signature of a loosely coupled system. When a system is complex and tightly coupled, NAT states that accidents are inevitable (i.e., normal). A discussion of NAT is not provided in this work because a technique for incident analysis based on NAT has never been developed. Besides, safety researchers have advocated for a more detailed view of accidents to be used.^18^, ^60^, ^61^ For example, different types of complexity and coupling exist, and such information is used to specifically engineer systems to be safe. These nuances are part of the reason why some systems classified as highly complex and tightly coupled (e.g., nuclear weapon system) actually experience very low accident rates. Nonetheless, NAT has fundamentally shaped safety thinking for many, especially on the topic of complexity. Furthermore, it has directly influenced safety improvement in radiation oncology.^62^, ^63^

Functional Resonance Analysis Method (FRAM) and Bowtie Analysis are other existing tools that have been used for safety improvement but are not reviewed in this work because they are not based on a specific accident model. FRAM is a method for system analysis with a focus on process functions and how they depend on one another.^64^ Each function is characterized in terms of six attributes: (1) input, (2) output, (3) precondition (necessary state before the function can begin), (4) resource, (5) control (instructions for execution), and (6) time (temporal considerations for execution). One of the principles underlying FRAM is that functions can interact to produce outsized variability in outcome, that is, functional resonance. The relevance to safety is that functional resonance can lead to an accident and thus needs to be prevented. Understanding functions in terms of the mentioned attributes can inform how the functions may interact. Strictly speaking, FRAM is not based on an accident model, and its intended use goes beyond prospective and retrospective safety analyses.^65^ While FRAM can be applied for incident analysis, that use is less common.^66^, ^67^ More experience of FRAM application remains to be gathered in healthcare, and opportunities for further development exist.^68^

Bowtie Analysis has been applied to understand incidents through a diagram shaped like a bowtie.^67^ The bowtie diagram is anchored on a top event where control is lost (center of the bowtie). To the left of the top event are lines tracing the top event to other preceding events that can cause the top event. This tracing graphically forms the left half of a bowtie. On the right‐hand side of the top event, lines fan out to depict a variety of consequences, which then completes the shape of the bowtie. Barriers that mitigate either the causes or the consequences are also depicted with symbols overlaid on the lines. Overall, Bowtie Analysis may be useful to communicate safety learning and as a summary of ideas generated, but its creation is based on other analyses tied to the chain‐of‐event models. Detailed recommendations for its general usage are provided elsewhere.^67^

## 7 SUMMARY

Safety can be effectively improved using a variety of analysis techniques. The VA RCA process and the London Protocol were developed to maximize incident learning based on a chain‐of‐event accident model and can be effective for incident analysis. Newer accident models (RMF and STAMP) and related analysis techniques (AcciMap and CAST) have been specifically developed for application to complex systems. Focusing on the contextual factors and component interactions can elicit meaningful explanations for actions or inactions whose safety impact may only be apparent after the fact. Where resources are available, expanding the analysis to systematically examine the control relationships between the frontline components and the organizational elements is important for eliciting areas of improvement for management, vendors, regulatory bodies, and professional associations. Taking a systems approach requires an effort to learn and adopt a different mindset from the linear thinking embodied by the chain‐of‐event models. The transition may be facilitated by the resources tailored to the healthcare community. Additional resource development and application experience sharing would also be valuable.

## AUTHOR CONTRIBUTIONS

Lawrence Wong and Todd Pawlicki jointly performed conceptualization, investigation, writing, and visualization of the work and agreed to be accountable for all aspects of the work.

## CONFLICT OF INTEREST STATEMENT

Lawrence Wong and Todd Pawlicki received research funding from Varian Medical Systems. Todd Pawlicki has received speaking honoraria from Varian Medical Systems and is a founding partner of Image Owl, LLC.
